# White Stork Pellets: Non-Invasive Solution to Monitor Anthropogenic Particle Pollution

**DOI:** 10.3390/toxics12040236

**Published:** 2024-03-23

**Authors:** Dora Bjedov, Alma Mikuška, Vlatka Gvozdić, Petar Glavaš, Dora Gradečak, Mirta Sudarić Bogojević

**Affiliations:** 1 Croatian Institute for Biodiversity, BIOTA Ltd., 10000 Zagreb, Croatia; dora.bjedov@gmail.com; 2 Department of Biology, Josip Juraj Strossmayer University of Osijek, 31000 Osijek, Croatiamsudaric@biologija.unios.hr (M.S.B.); 3 Department of Chemistry, Josip Juraj Strossmayer University of Osijek, 31000 Osijek, Croatia

**Keywords:** regurgitated pellets, anthropogenic particles, pollution monitoring, dietary assessment

## Abstract

The present study applied a non-invasive method to analyse anthropogenic particles and prey items in white stork (*Ciconia ciconia*) pellets. Pellets (*n* = 20) were obtained from white stork nests during the 2020 breeding season from two sites in Croatia. In total, 7869 anthropogenic particles were isolated. The majority of particles were fragments, while previous studies on other birds often reported fibres. An ATR–FTIR polymer analysis detected glass and construction and building materials, as well as several compounds associated with plastic masses. Polymer investigation revealed the presence of dotriacontane and octacosane, which are by-products of polyethylene (PE) degradation and transformation. Additionally, the detection of vinylidene chloride (VDC) highlights the historical contribution of polyvinylidene chloride (PVDC) to plastic pollution. Significant variation in particle quantity and size between the sampling sites was detected, with larger particles found at sites associated with the metal mechanical engineering industry and agriculture. Prey assessment revealed chitin remains of large insects such as Orthoptera and Coleoptera. This research confirms the potential of pellet analysis as a valuable tool for assessing the presence of anthropogenic particles in the environment. However, further research is needed to fully understand the extent of particle ingestion, particle sources and potential impact.

## 1. Introduction

Emerging pollutants comprise a wide category of dangerous substances, such as nanomaterials, nanoplastics, microplastics, soot and wear from roads and tyres. These anthropogenic particles are produced by human activities, resulting in their broad spatial range [1]. They are manufactured in millions of metric tonnes per year and can be released into the environment, potentially causing adverse effects on biota, the environment and public health [2]. Awareness and interest in their potentially harmful consequences have increased, especially for those at the micro- and nanoscale, e.g., organic and inorganic anthropogenic fragments [3,4]. Once in the environment, anthropogenic particles degrade into smaller particles via biotic and abiotic mechanisms, e.g., biodegradation, photodegradation, oxidation and/or abrasion [5].

Research regarding anthropogenic particle pollution has been primarily focused on the aquatic system, mainly regarding the transfer of anthropogenic particles through food webs and their effects on apex predators [6,7]. Anthropogenic particle ingestion has been previously investigated in aquatic systems via aquatic bird species, both marine (e.g., Cassin’s auklet, *Ptychoramphus aleuticus* [8] and little auks, *Alle alle* [9]) and freshwater (e.g., Clapper rails, *Rallus crepitans* and Seaside sparrows, *Ammospiza maritima* [10]). However, recent studies have shown that anthropogenic particle pollution is a current ubiquitous issue [11,12]; therefore, advances have been made by analysing plastic particles in terrestrial birds. Several studies have assessed the environmental burden of anthropogenic particles in terrestrial ecosystems via white stork carcasses, focusing on general plastic ingestion [13], rubber band ingestion [14] and ingestion of plastic objects due to feeding at urban refuse dumps [15]. An additional aspect of monitoring could be accomplished by examining the quantity of anthropogenic materials utilised in the nest construction, as they can exhibit a correlation with the degree of urbanisation [16,17]. The incorporation of anthropogenic materials into nests could be affected by mating behaviour as well. Bowerbirds (Ptilonorhynchidae) construct bowers to allure potential mates [18]. The decoration of bowers plays a pivotal role in female mate selection, with bowerbirds embellishing their bowers with a variety of items, including flowers, plants and human debris such as bottle tops and straws [18]. Males with more elaborately decorated bowers are deemed more attractive and enjoy enhanced reproductive success, potentially leading to an increase in the prevalence of anthropogenic materials within bowers [18]. On the other hand, an aspect of the negative effects of anthropogenic particles was investigated, namely, the occurrence of anthropogenic materials in white stork nests, which are often associated with better breeding success. However, on the other hand, a higher risk of nestling mortality is possible due to ingestion and/or entanglement of particles [19]. Apart from lethal effects, as previously described, anthropogenic particles can cause sublethal effects, reflected in an increase in oxidative stress, overall redox imbalance and cholinesterase activity [20]. Monitoring of anthropogenic particles and their possible effects as well as integrated biomarker assessment have been used in Japanese quail, *Coturnix japonica* [20], common blackbird, *Turdus merula*, song thrush, *Turdus philomelos* [21] and tree swallow, *Tachycineta bicolor* [22], indicating the use of the aforementioned species as bioindicators of anthropogenic particle pollution in terrestrial ecosystems.

Monitoring strategies for anthropogenic particles as alternatives to bird remains include their undigested prey residues—regurgitated pellets. Pellet analysis provides information regarding prey composition as well as the occurrence of anthropogenic particles. A species that regurgitates pellets and is representative of the terrestrial ecosystem is the white stork, *Ciconia ciconia*. The species is distributed in continental Croatia [23], with opportunistic dietary habits, feeding predominantly on earthworms, grasshoppers, fish, frogs and small mammals [24]. Foraging near landfills has also been recorded [25,26,27]. White storks are diurnal predators, with habitat preferences in open lands, e.g., agricultural areas, wet grassland and arable lands [28]. Breeding white storks are conservative in their habitat selection, with significantly smaller home ranges, when compared to non-breeding white storks [29]. Moreover, white storks have low reproductive dispersal and usually return to the same nest as in previous years [30]. Therefore, the content of anthropogenic particles in the pellets could reflect the local environmental burden and trophic transfer.

The present research considered the white stork pellets by reporting qualitative and quantitative analysis of anthropogenic particles and fibres (plastics, textiles, construction and demolition waste and glass) in pellets from white storks. Although white stork pellets have been used for investigation to quantify their exposure to indigestible litter of anthropogenic sources and diet assessment [31], the novel aspect of this research is reflected in polymer analysis of the isolated anthropogenic particles. Therefore, the objectives of the research are as follows:

(I) Investigate the application of white stork pellets for anthropogenic particle monitoring. Since the white stork is an undomesticated species that is ecologically associated with urban settlements, their habits (behavioural and dietary) could potentially make them effective indicators of micro-anthropogenic particle pollution caused by anthropogenic activities;
(II) Perform polymer analysis on suspected anthropogenic and other non–biological particles;
(III) Examine if there is a spatial variation in the number of micro-anthropogenic particles isolated, as the assumed polluted sampling site is an area surrounded by a major river, industry and agricultural land, and is adjacent to the urban centre;
(IV) Investigate the prey composition of pellets to determine the prevalence of food sources and feeding habits of white storks in sampling locations.

## 2. Materials and Methods

### 2.1. Sampling Locations

**Figure 1:**
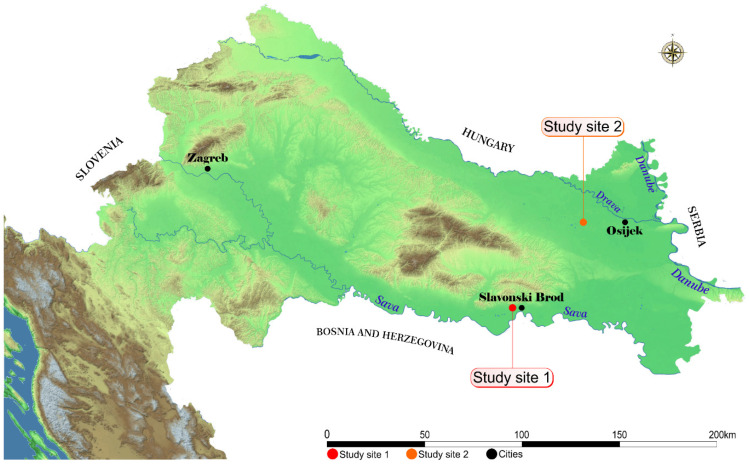
The geographical location of sampling sites.

Regurgitated pellets were obtained from white stork nests during the breeding season in June and July 2020. In total, 20 pellets were collected and analysed from two sampling areas (Figure 1). Each pellet represented one nest. Pellets from selected nests for sampling in Study Site 1 (*n* = 10) lay along the Sava River, just downstream from an urban centre (Slavonski Brod) known for its highly developed metal engineering industry. The nests are surrounded by agriculture, small villages, alluvial forests and pastures regularly flooded by Sava. Furthermore, an oil refinery is situated at Bosanski Brod, which is adjacent to the town of Slavonski Brod. Pellets sampled from nests in Study Site 2 (*n* = 10) were located in small villages, surrounded by large grassland pastures, meadows, arable land and woodland habitat.

### 2.2. Isolation and Analysis of Anthropogenic Particles

Following the field sampling, all pellets were kept at −20 °C to prevent microbial growth until analysis. Thawed pellets were weighed and dissected. Potential anthropogenic particles were visually detected with a high-quality stereomicroscope Leica MZ6 and categorised by size into microanthropogenic (< 0.50 mm) and macroanthropogenic particles (>0.50 mm). Another category of the particles was shape (e.g., fragment, filament). Suspected isolated particles were subsequently corroborated with the hot needle method. The hot needle method has been used as the visual verification prior to advanced polymer identification. To expand, a histological needle was heated on a glass alcohol burner and put on the suspected particle of anthropogenic origin. A positive response was observed if the particle melted or curled, rather than charred [32,33]. The detection of microplastic particles smaller than 0.5 mm was performed based on shape and colour with an optical microscope (Leica MZ6). The isolated micro– and macroanthropogenic particles were transferred to glass vials with metal tweezers and stored until analysis.

### 2.3. Spectroscopic Analysis

Polymer analysis of isolated particles was performed with attenuated total reflection Fourier transform infrared spectroscopy (ATR–FTIR). In total, 642 particles were selected for analysis based on the hot needle test, size, shape and colour. Anthropogenic particles were analysed with ATR–FTIR in a wavenumber range of 4000–450 cm^−1^. Each sample was measured in six technical replicates. The obtained spectrum for each sample was recorded as % transmittance (T) using a Perkin-Elmer Spectrum Two with Universal ATR, controlled by the software Spectrum 10.5.2.636.

### 2.4. Prey Remains Isolation and Determination

In parallel with anthropogenic particles, prey remains were isolated with dry method pellet analysis, according to Horváth et al. [34]. The identification of prey was based on the morphological characteristics of the remains. Prey items were identified at the lowest possible taxonomic level. Chitinous pieces of insects were identified according to Chinery [35] and by comparison with entomological collections of species commonly present in the studied areas.

### 2.5. Quality Control

Quality control precautions were implemented during the isolation and polymer analysis of anthropogenic particles. Plastic materials were intentionally avoided throughout the process of pellet collection, sample isolation and sample analysis. Instead, preference was given to the use of glass vials and Petri dishes, as well as aluminium and stainless-steel utensils, for all equipment. Additionally, lab coats and nitrile gloves were worn, samples were covered with aluminium foil when not being used or processed and procedural blanks were used. Particles were isolated in a laminar flow cabinet equipped with vertical HEPA filters (MINIFLO Type 90, Milan, Italy). The laboratory workspace as well as tweezers, needles, glass vials and Petri dishes were meticulously cleaned with 70% ethyl alcohol.

### 2.6. Statistical Analysis

Statistical tests were performed using R version 4.2.2 and Statistica version 14.0.0.15. To identify the patterns and/or trends in the data that may indicate variations in polymer composition with regard to sampling sites, principal component analysis (PCA) was performed. To compare the number of isolated anthropogenic particles with regard to sampling sites, the number of particles per mass of the pellet (n_particle_ g_pellet_^–1^) was used. To test the normality of the data distribution, the Shapiro–Wilks test was applied. Data were not normally distributed; therefore, the non-parametric, unpaired, two-tailed Mann–Whitney U test was applied by comparing the ranks. The level of statistical significance (*p*-value) was 0.05 throughout the study.

## 3. Results

### 3.1. Isolated Anthropogenic Particles

**Figure 2:**
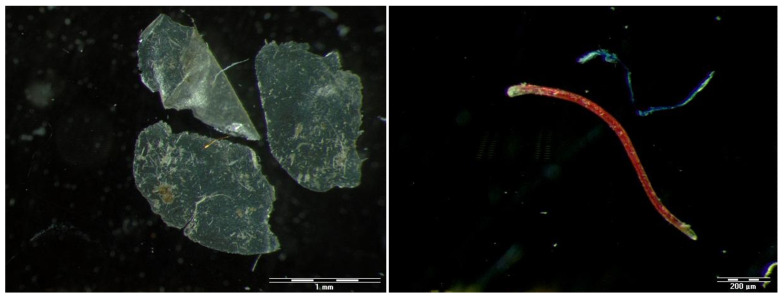
The most common anthropogenic particles found in white stork (*C. ciconia*) pellets were clear fragments (**left**; compound dotriacontane) and coloured filaments (**right**; compound paraffin oil).

Anthropogenic particles were detected in all analysed pellets. Particles such as microplastic fragments, filaments, building materials and glass were isolated and morphological characteristics were determined. More than 90% of anthropogenic particles were clear fragments, followed by filaments (Figure 2). Microanthropogenic particles were detected in all pellets, while macroanthropogenic particles were reported in 60% of analysed pellets.

### 3.2. ATR–FTIR Results of Analysed Particles

**Figure 3:**
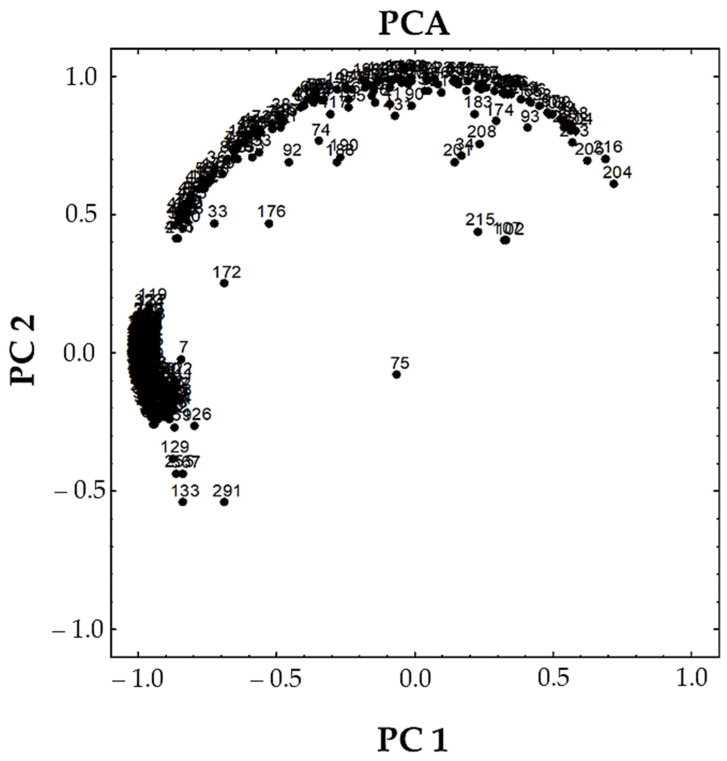
The results of principal component analysis (PCA): one complex cluster of all analysed spectra.

We detected substances associated with plastic masses, which are shown in Table 1. Out of 7869 isolated particles, we detected polymers associated with plastic masses in 519 particles, namely, in Study Site 1, 321 (4.23%) particles and, in Study Site 2, 198 (49.38%) were associated with plastic masses. PCA analysis was performed on 499 spectra. The results showed similarities among samples collected from different locations. The results of PCA showed that two principal components account for 80% of the total variance in the data (Figure 3).

### 3.3. Spatial Variability

In total, 7869 micro- and macroanthropogenic particles were isolated from the pellets. An overview of the results is shown in Table 2. In particular, there were 7869 particles in Study Site 1 and 401 in Study Site 2, confirming the significant spatial variation in particle quantity (*p* < 0.0001). Regarding particle size, significantly larger particles were detected in Study Site 1 compared to Study Site 2 (Table 2).

### 3.4. Dietary Assessment

Pellet analyses showed that white storks from study locations fed on insects (Insecta), spiders (Arachnida), snails (Gastropoda), earthworms (Clitellata) and mammals (Mammalia). In all analysed pellets, remains of mammals’ hair and earthworms’ chaetae were found (Table 3), along with different blades of grass and other plants. Among insects, the most abundant prey remains belonged to beetles (Coleoptera), grasshoppers, locusts and crickets (Orthroptera). Differences between prey remains from the two study sites are presented in Table 3.

## 4. Discussion

The present study implemented the method of collecting and analysing white stork pellets for the purpose of anthropogenic particle monitoring. Among species that regurgitate pellets, anthropogenic and plastic particles have been detected in white stork, kingfisher, *Alcedo atthis* and barn owl (*Tyto alba*) [31,36,37]. That being said, our results correspond with the study by Mikula et al. [26], as we detected anthropogenic particles in all analysed pellets as well. Anthropogenic particles were also detected in earlier examinations of white stork pellets collected in Bulgaria during the non-breeding season, albeit at far lower frequencies in pellets for glass (2.7%) and plastic (4.1%) [38]. Nessi et al. [37] analysed microplastics in the pellets of a nocturnal bird of prey, barn owl. The authors associated the microplastic from the pellets with prey due to degradation of habitat, i.e., agricultural lands [37]. In research on kingfisher, a piscivore top predator in river ecosystems, the authors suggested that the ingestion was more likely derived from their food rather than from abiotic elements such as sediment and water [36]. Research on waterbirds suggests the ingestion of microplastics likely originates from sediment particles and water rather than from their food, although this has yet to be conclusively proven [39]. Regarding shape, most detected particles from other studies were fibres [36,37,40], while, in the present study, most detected particles were fragments. Anthropogenic micro–fragments can be derived from the breakdown, fragmentation or degradation of larger anthropogenic particles [41]. Although results from the present study are difficult to compare to other studies due to different avian foraging strategies, pellet regurgitation, habitat, research methodology and pollutant accumulation, continuous detection of anthropogenic particles in pellets, digestive tract and faeces indicates environmental pollution, warranting design of mitigation measures. When interpreting results, several sources of anthropogenic particles in pellets should be taken into account. For example, particles can be ingested primarily by accident together with smaller food items such as insects or secondarily if the anthropogenic particles are digested by their prey. An additional source of particles in sampled pellets could be atmospheric deposition [42].

PCA results did not show any significant clustering of the polymer compounds based on the sampling site variable. Anthropogenic pollution appears similar in a polymer sense but differs in quantity, as seen by the number of isolated particles per site. According to Moore [43], the polymers found in microplastic pollutants can undergo degradation and possible chemical changes due to exposure to the environment. Furthermore, Lundquist et al. [44] suggest that microplastic pollutants consist of various inorganic fillers, plasticisers and UV stabilisers, which may also undergo alterations caused by environmental conditions. The ATR–FTIR spectra of a microplastic particle will reflect all the chemical changes it has experienced, including the presence of non–polymer compounds from the pollutant. However, it is crucial to consider the presence of typical additives and co–polymers that might also be present when interpreting the results.

The most common chemical compounds when analysing isolated microparticles were dotriacontane and octacosane. According to Abraham et al. [45], dotriacontane is a by-product of plastic polymer polyethylene (PE) degradation by fungi, *Aspergillus nomius*. Octacosane and 1,3,5-trimethylcyclohexane are by-products of low-density PE transformation under high temperatures [46,47]. Since PE is a polymer that is primarily used for packaging, e.g., plastic bags, films and containers, this represents the first association with microplastic particles in white stork pellets. Several other compounds associated with plastic masses have been detected. Compound 3-(2-imidazolin-1-YL) propyltriethoxysilane is used in resin and plastic production [48] and methyl linoleate is a plasticiser used for polyvinyl chloride (PVC) [49]. Enzacryl polyacetal is a synthetic polymer, a thermoplastic used in engineering. Previously, it has been characterised only in aquatic ecosystems, namely, two fish: Spotted Tail goby, *Synechogobius ommaturus*, and Seabass, *Lateolabrax japonicus* [6]. Another thermoplastic compound detected was (3-aminopropyl)triethoxysilane. Additionally, we detected vinylidene chloride (VDC, 1,1-dichloroethylene), a compound used in the production of the polymer polyvinylidene chloride (PVDC). PVDC is well known for its barrier properties and is used extensively as a coating for various packaging materials, especially in the food industry. It is often used in combination with other polymers to create materials with enhanced barrier properties against moisture, oxygen and other gases [50]. While PVDC itself is not as commonly used today due to environmental and health concerns related to the release of vinyl chloride monomer during production and incineration, it has historically been a significant contributor to plastic pollution [51]. Polymer analysis revealed paraffin oil on the analysed particles. Paraffin oil has many uses in the plastic industry and is associated with agriculture, e.g., petroleum-based insecticides and as a part of diesel fuel for tractor engines [52]. White storks are frequently associated with foraging on arable lands; therefore, it is no surprise the residues of agricultural and farming equipment have been detected. Chemicals obtained by bacterial degradation of chlorinated paraffins were observed. Dioctyl sebacate and 1-chlorohexadecane are examples of additives used in plastics to modify certain properties or facilitate the manufacturing process, namely, 1-chlorohexadecane was detected and its main purpose is industrial. It is frequently added in plasticisers and flame retardants [53]. Apart from chemicals associated with plastic degradation, compounds (hexacosanol) used in plastic production as molecular lubricants for plastic polymers were detected [54]. In particular, butyl stearate is used as a functional additive, acting as a lubricant in the plastic polymer polystyrene (PS). Volatile organic compounds (VOCs; e.g., 3-methylheptane) have been detected. VOCs are usually released in the environment by photodegradation of various plastic polymers, such as PS [55]. As previously mentioned, visual inspection of macroanthropogenic particles showed construction and building materials in the pellets. This was additionally confirmed by ATR–FTIR analysis of particles that contained octadecylamine. Octadecylamine is a compound associated with the improvement of the hydrophobic properties of polyurethane (PU) foam for the purpose of oil spill clean-up [56]. Ethyl palmitate was detected as well. The compound is a degradation product of PU [57]. Hydrocarbons were detected in the pellets as well. Hexatriacontane indicates the presence of these persistent organic pollutants (POP) derived from petroleum and contributes to environmental pollution and adverse effects on biota [58,59]. Potential sources of hexatriacontane are motorised activities and the petrochemical industry [58,60].

Anthropogenic particles obtained from regurgitated pellets from white storks’ nests at Study Site 1 and Study Site 2, varied significantly in particle quantity (Table 1). Regarding particle size, significantly larger particles were detected in Study Site 1 compared to Study Site 2 (Table 1). The white stork forages on open grasslands and floodplains, habitats often transformed into agricultural and farming lands. Agricultural soils may become long–term ‘sinks’ and reservoirs for anthropogenic particles [61,62]. This indicates that agricultural areas are vulnerable to pollution, reflected in anthropogenic particle detection in both study areas. However, a greater number of (and larger) man-made particles were detected in pellets from Study Site 1. We assume that the city and the urban residential area actively contribute to the anthropogenic particle pollution, based on the fact that microplastic particles have been detected in soil and surface road dust in urban cities [63,64]. Since the foraging area is in proximity to the urban centre of Slavonski Brod, the wastewater treatment plant (WWTP) in Slavonski Brod can be a potential source of anthropogenic particles via the release of effluent plants [65]. Furthermore, the metallurgic industry in Slavonski Brod and the oil refinery in Bosanski Brod could be major potential sources of pollution in the Sava River and the surrounding soil.

White storks regurgitate pellets daily or even more times per day, depending on prey abundance [66]. Foraging flights of the majority of white storks are within 1.5 km of nests [67,68], but foraging radius can be up to 5 km from nests [69]. The diversity of prey items depends on the conditions prevailing in their habitats—if the habitats are dry and there is no larger prey available, white storks will feed on insects [70]. Depending on the type of prey, white storks have different hunting strategies. They catch their prey with their long beaks, and, if it is a larger animal, they first kill it with a beak strike and then tear it apart. Insects are collected by searching through low vegetation [66]. In the dietary assessment, we found only small mammal hairs (from which it is not possible to determine species, number of specimens or their size) and no remains from fishes, amphibians or reptiles. Studies of white stork feeding habits show that the deficiency of prey remains of mammals, amphibians, reptiles or fishes in pellets does not reflect a lack of them in the feeding habitats, but rather that their remains are almost entirely digested [68,70]. We found numerous chitin remains of large insects—mandibles from Orthoptera and elytrons from Coleoptera. Our results comply with diet studies in Europe showing that insects are important prey for white storks, especially in southern parts of Europe where habitats are drier [71,72,73].

## 5. Conclusions

The present research successfully applied the pellets of an opportunistic terrestrial apex predator for anthropogenic particle monitoring. The findings suggest that pellet analysis offers a non–invasive method to assess the presence of various pollutants in the environment while reducing disturbance and minimising ethical concerns. Following a polymer analysis, we detected construction and building materials, glass and several compounds associated with plastic masses. The ATR–FTIR analysis of isolated particles revealed the presence of dotriacontane and octacosane, which are by-products of PE degradation and transformation. Additionally, the detection of VDC highlights the historical contribution of PVDC to plastic pollution. Regarding quantity, spatial variation was confirmed, as a higher number of fragments was detected from pellets in Study Site 1. It is assumed that the wastewater treatment plant in Slavonski Brod contributes to the high number of fragments. Diet assessment of the white stork revealed a lack of identifiable remains from fishes, amphibians or reptiles, suggesting efficient digestion, while chitin remains of large insects such as Orthoptera and Coleoptera were abundant. To conclude, the presence of man-made fragments in white stork pellets highlights the problem of widespread anthropogenic particles in the environment. By analysing the composition and characteristics of the particles found in the pellets, it is possible to identify specific pollutants, their origins and pollutant hotspots, making storks valuable indicator species for environmental monitoring. Analysis of pellets over time offers a valuable means to elucidate temporal variations in pollutant concentrations and trends, thereby facilitating a comprehensive understanding of pollution dynamics within the ecosystem. Such insights are instrumental in informing, formulating and refining policies and regulations that are targeted at mitigating particle pollution, ultimately contributing to environmental management and public health enhancement efforts. Additionally, the chemical compounds associated with anthropogenic and plastic debris and the analysis of anthropogenic particles (as well as microplastics) should be considered in future research to understand their effect on biota and their role in the ecosystem, if any.

## Figures and Tables

**Table 1:** Results of polymers detected with ATR–FTIR. For each polymer, a use was described as well as whether it is associated with plastic masses.

Polymer	Uses	Associated with Plastic Masses
(3-aminopropyl)triethoxysilane	thermoplastic polymer	yes
1,2-octadecanediol	personal care products	no
1,3,5-trimethylcyclohexane	by-product of PE	yes
1-chlorohexadecane	additive used in plastic production	yes
3-(2-imidazolin-1-YL)propyltriethoxysilane	resin and plastic production	yes
3-methylheptane	product of PS degradation	yes
Butyl stearate	additive used in plastic production	yes
Dioctyl sebacate	additive used in plastic production	yes
Dotriacontane	by-product of PE	yes
Enzacryl polyacetal	thermoplastic polymer	yes
Ethyl palmitate	product of PU degradation	yes
Hexacosanol	plastic production	yes
Hexatriacontane	petroleum product	no
L(-)-glyceraldehyde unnatural forms	naturally occurring	no
Methyl linoleate	PVC plasticiser	yes
Octacosane	by-product of PE	yes
Octadecylamine	product of PU degradation	yes
Paraffin oil	plastic production	yes
Polystyrene	plastic polymer	yes
Tetradodecylammonium bromide	surfactant and catalyst	no
Toluene-4-sulfonic acid	surfactant and catalyst	no
Vinylidene chloride	plastic production	yes

**Table 2:** Number, mass and diameter of isolated anthropogenic particles from white stork (*C. ciconia*) pellets sampled during breeding season 2020 in continental Croatia.

	n_particle_	Mass (g)	n_particle_ g_pellet_^–1^	Min	Max	Mean ± SD
Study Site 1 (n_pellet_ = 10)	284	13.23	21.47	<0.50	20.00	2.54 ± 1.68
	239	6.25	38.22	1.00	40.00	2.27 ± 3.10
	33	11.26	2.93	1.00	10.00	3.12 ± 1.68
	105	12.28	8.55	1.00	10.00	2.10 ± 1.21
	86	12.28	7.00	<0.50	10.00	2.37 ± 1.30
	660	9.00	73.37	1.00	22.00	2.39 ± 0.93
	1411	27.93	50.51	<0.50	13.00	2.33 ± 0.93
	796	7.20	110.49	<0.50	7.00	2.02 ± 0.93
	1996	22.08	90.39	<0.50	20.00	1.80 ± 1.29
	1858	13.51	137.51	<0.50	12.00	2.32 ± 1.26
Study Site 2 (n_pellet_ = 10)	51	9.63	5.30	<0.50	5.00	1.37 ± 0.91
	27	10.73	2.52	<0.50	3.00	1.28 ± 0.71
	35	17.16	2.04	<0.50	1.00	0.73 ± 0.24
	33	7.53	4.38	<0.50	2.25	0.93 ± 0.46
	125	11.51	10.86	<0.50	4.25	1.32 ± 0.83
	12	8.24	1.46	<0.50	1.20	0.76 ± 0.22
	12	4.80	2.50	<0.50	2.50	1.25 ± 0.58
	4	11.87	0.34	<0.50	1.20	0.85 ± 0.31
	9	8.95	1.01	<0.50	35.00	5.83 ± 11.23
	93	7.23	12.87	<0.50	9.00	1.84 ± 1.46

n_particle_—number of isolated anthropogenic particles; Mass—the mass of the whole dry pellet; n_particle_ g_pellet_^–1^—number of isolated anthropogenic particles per gram of the pellet; Min—minimum diameter of particles in the pellet; Max—maximum diameter of particles in the pellet.

**Table 3:** Taxonomic groups of prey items determined in the pellets of white stork (*C*. *ciconia*) and their occurrence in Study Site 1 and Study Site 2.

Class	Order	Family	Species	Study Site 1	Study Site 2
Mammalia	Rodentia			x	x
Arachnida	Araneae			x	
Clitellata	Opisthopora	Lumbricidae		x	x
Mollusca	Gastropoda		*Gastropoda terrestria* sp.	x	
Insecta	Diptera				x
	Hymenoptera	Formicidae		x	
	Orthoptera	Gryllidae		x	x
		Tettigoniidae			x
		Acrididae			x
		Gryllotalpidae	*Gryllotalpa gryllotalpa*	x	x
	Coleoptera	Chrysomelidae		x	
		Silphidae/		x	x
		Lucanidae	*Dorcus parallelipipedus*	x	x
		Cerambycidae			x
		Tenebrionidae	*Blaps mortisaga*	x	
		Scarabaeidae	*Melolontha* sp.		x
			*Melolontha melolontha*	x	
			*Oryctes nasicornis*	x	
			*Cetonia aurata*	x	
		Carabidae	*Carabus* sp.	x	x
			*Abax* sp.	x	x
			*Calosoma* sp.		x
			*Harpalus* sp.		x
			*Abax* sp.	x	x
			*Carabus ullrichi Germar*	x	x
			*Carabus granulatus*	x	
			*Carabus violaceus*	x	
			*Carabus coriaceus*	x	
			*Carabus intricatus*	x	
			*Calosoma auropunctatum*	x	

## Data Availability

All data are included in the manuscript.
